# MiR-137-mediated negative relationship between *LGR4* and *RANKL* modulated osteogenic differentiation of human adipose-derived mesenchymal stem cells

**DOI:** 10.1590/1678-4685-GMB-2021-0332

**Published:** 2022-09-19

**Authors:** Cong Fan, Yulong Li

**Affiliations:** 1 Peking University School and Hospital of Stomatology, Department of General Dentistry II, Beijing, China.; 2 National Center of Stomatology, Beijing, China.; 3 National Clinical Research Center for Oral Diseases, Beijing, China.; 4 National Engineering Research Center of Oral Biomaterials and Digital Medical Devices, Beijing, China.; 5 Health Service Department of the Guard Bureau of the General Office of the Central Committee of the Communist Party of China, Beijing, China.

**Keywords:** MicroRNA, LGR4, osteogenic differentiation, human adipose-derived mesenchymal stem cells, RANKL

## Abstract

MicroRNA-137 (miR-137) has recently emerged as an osteogenic regulator in several cell lines. This study aimed to identify the function of miR-137 on the crosstalk between *leucine rich repeat containing G protein-coupled receptor 4* (*LGR4*) and *receptor activator of nuclear factor-*κ*B ligand* (*RANKL*), thus unveiling the critical role of *LGR4*-*RANKL* interplay in the osteogenic differentiation of human adipose-derived mesenchymal stem cells (hASCs). By examining the osteogenic capacity and possible downstream genes expression with miR-137 overexpression/knockdown, we found that miR-137 downregulated *LGR4* while upregulating *RANKL*. According to the results of dual-luciferase reporter assay, *LGR4* was validated as a direct target of miR-137. Surprisingly, a negative relationship between *LGR4* and *RANKL* was confirmed by the knockdown of these two genes. Furthermore, *RANKL* inhibitor could alleviate or reverse the inhibitory effects on osteogenesis generated by *LGR4* knockdown. Collectively, this study indicated that miR-137-induced a negative crosstalk between *LGR4* and *RANKL* that could contribute to the osteogenic regulation of hASCs and provide more systematic and in-depth understanding of epigenetic modulation by miR-137.

## Introduction

As an ideal stem cell source, human adipose-derived mesenchymal stem cells (hASCs) are widely used in bone tissue engineering due to their accessibility, multipotency and low immunogenicity (Dinescu *et al.*, 2021). MicroRNAs (miRNAs), a class of small non-coding RNAs ranging from 19-25 nucleotides in size, regulate protein-coding genes post-transcriptionally and play crucial roles in various biological activities, including the proliferation and osteogenic differentiation of hASCs (Ranganathan and Sivasankar, 2014; Fan *et al.*, 2021). It has been demonstrated that miR-137 acts as a key regulator in various kinds of malignancies (Li *et al.*, 2017; Liu *et al.*, 2017; Qin *et al.*, 2017; Luo *et al.*, 2018; Zhang *et al.*, 2018) and neural development (Silber *et al.*, 2008; Szulwach *et al.*, 2010; Tarantino *et al.*, 2010; Sun *et al.*, 2011; Jiang *et al.*,2013). But up to present, only a few studies investigated the effects of miR-137 on osteogenesis (Zheng *et al.*, 2019; Kong *et al.*, 2020; Ma *et al.*, 2020; Yu *et al.*, 2020; Fan *et al.*, 2021), and the mechanisms of osteogenic modulation by miR-137 are still not fully understood, especially considering the diverse biological properties of different cell types.

The gene *Leucine rich repeat containing G protein-coupled receptor 4* (*LGR4*) has been implicated in various biological processes, such as bone mineralization and remodeling, innate immune responses, intestinal stem cell metabolism and energy metabolism (Zhou *et al.*, 2017). Some studies have confirmed the direct binding of *LGR4* by miR-137 in prostate cancer cells (Zhang *et al.*, 2020), U-2 and MC3T3 cells (Liu and Xu, 2018). However, whether *LGR4* is a target of miR-137 in hASCs needs to be identified. Moreover, *LGR4* is found to be engaged in the regulation of osteogenesis, and its inhibition leads to impaired bone formation in several lineages of mesenchymal stem cells (Zhang *et al.*, 2017; Zhou *et al.*, 2017; Sun *et al.*, 2019). Based on these findings, we conjectured that miR-137 could regulate the osteogenic differentiation of hASCs through *LGR4*.

The gene product of *Receptor activator of nuclear factor-κB ligand* (*RANKL*) is membrane-bound or secreted by either proteolytic cleavage or alternative splicing (Chen *et al.*, 2018a). After binding with Receptor activator of nuclear factor-κB (RANK), RANKL activates the downstream signaling pathways related with osteoclastic proliferation and differentiation (Boyle *et al.*, 2003). Besides, RANKL can positively regulate the osteoblastic differentiation of vascular smooth muscle cells (VSMCs) (Davenport *et al.*, 2016), while negatively regulating the osteoblastic differentiation of bone marrow mesenchymal stem cells (BMSCs) (Chen *et al.*, 2018b; Elango *et al.*, 2019), suggesting that RANKL could have effects on the osteogenic differentiation of hASCs.

Recent studies have shown that LGR4 is a novel receptor of RANKL and negatively regulates osteoclast differentiation by the competition with RANK to bind to RANKL (Luo *et al.*, 2016; Renema *et al.*, 2016). But the silencing of *LGR4* in VSMCs can prevent parathyroid hormone (PTH)-induced vascular calcification without changes in *RANKL* and *OPG* expression (Carrillo-López *et al.*, 2021). However, how the interplay of LGR4-RNAKL influences the osteogenic differentiation of hASCs is not clear so far. Therefore, we proposed as a hypothesis that miR-137 regulates the osteogenesis of hASCs by mediating the interplay between LGR4 and RANKL.

In this study, we confirmed that *LGR4* was a target gene of miR-137 in hASCs and the crosstalk between *LGR4* and *RANKL* participated in the osteogenic control of miR-137. The revelation of epigenetic mechanisms on miR-137-modulated osteogenesis is valuable for miRNA-targeted therapy of bone defect, and the findings of LGR4-RANKL contribution in the osteogenic differentiation has implications for future clinical management of bone disorders.

## Material and Methods

*Cell culture and osteogenic induction*

The hASCs collected from three different donors were obtained at ScienCell Research Laboratory (Carlsbad, CA) and cultured in proliferation medium (PM), which contained Dulbecco’s modified Eagle’s medium (Thermo Fisher Scientific, Rockford, IL), 10% (v/v) fetal bovine serum (ExCell Bio, Shanghai, China) and 1% (v/v) penicillin/streptomycin (Thermo Fisher Scientific). For the osteoinduction of hASCs, cells were cultured in osteogenic medium (OM), composed of PM, 100 nM dexamethasone (Sigma‐Aldrich, St. Louis, MO), 10 mM β‐glycerophosphate (Sigma-Aldrich) and 0.2 mM L‐ascorbic acid (Sigma-Aldrich). The hASCs were incubated at 37 °C with 5% CO_2_ and 100% humidity. All the *in vitro* cell experiments applied the third passage (P3) hASCs and were repeated in triplicate at least.

*Lentiviral transfection*

GenePharma (Suzhou, China) synthesized and packaged the recombinant lentiviruses containing green fluorescent protein (GFP)‐labeled plasmid vectors. The plasmids of negative control (NC), miR‐137 overexpression (miR‐137), miR‐137 knockdown (anti‐miR‐137), *LGR4* shRNA (anti-*LGR4*) and *RANKL* shRNA (anti-*RANKL*) were separately employed to generate the corresponding lentiviruses. The hASCs were transfected by respective lentiviruses as previously described (Ma *et al.*, 2020;Fan *et al.*, 2021). After being immersed in the viral supernatant (multiplicity of infection = 100) with 5 mg/mL polybrene (Sigma-Aldrich) for 24 h, the cells were then selected by 1 μg/ml puromycin (Sigma-Aldrich) and cultured in fresh PM. The transfected rates were computed by counting the number of GFP-marked cells and total cells under an inverted fluorescence microscope (TE2000-U, Nikon, Tokyo, Japan). The hASCs transfected with anti-*LGR4* lentiviruses were seeded in 96-well plates (1 × 10^4^ cells/well) and treated with *RANKL* inhibitor (denosumab; Amgen, Thousand Oaks, CA) at a concentration of 5 μg/ml before the examination of osteogenic ability.

*Alkaline phosphatase (ALP) staining and quantification*

The transfected hASCs were seeded at a density of 2 × 10^5^ cells/well in 24-well plates and cultured in PM or OM for seven days. ALP staining was performed following the instructions of BCIP/NBT staining kit (Beyotime, Shanghai, China). As for the quantification of ALP activities, cells were rinsed with phosphate buffer saline (PBS) and 1% Triton X-100 (Solarbio, Beijing, China) three times, then scraped into milli-Q water and underwent three freeze-thaw cycles. By applying the pierce BCA protein assay kit (Thermo Fisher Scientific), total protein was read at 562 nm and calculated with a bovine serum albumin standard curve according to the manufacturer’s protocol. Finally, ALP activity was tested at 520 nm using an alkaline phosphatase assay kit (Jiancheng, Nanjing, Jiangsu, China) and calculated after normalization to the total protein concentrations.

*Mineralization assays*

The extracellular mineralization level of hASCs were tested after a 14-day *in vitro* culture with PM or OM. After being fixed with 95% ethanol for 30 min, the cells were steeped in 1% alizarin red s (ARS) staining solution (pH 4.2; Sigma-Aldrich) at room temperature for 30 min. To quantify the degree of matrix calcification, ARS-stained plates were separately dissolved by 100 mM cetylpyridinium chloride (Sigma-Aldrich) for 1 h and then the absorbances were detected with an EnSpire multimode plate reader (PerkinElmer, Waltham, MA) at 562 nm. Finally, the ARS relative intensity was calculated after normalization to the total protein concentrations.

*Quantitative real-time polymerase chain reaction (qRT-PCR)*

The total RNA of hASCs was lysed in TRIzol (Invitrogen, Carlsbad, CA) and complementary DNA was synthesized using a reverse transcription system (Takara, Tokyo, Japan). The quantification of miR-137 and gene transcripts were respectively examined using the miScript SYBR Green PCR kit (Qiagen, Frankfurt, Germany) and FastStart universal SYBR green master (ROX) (Roche, Indianapolis, IN) on a 7500 real-time PCR detection system (Applied Biosystems, Foster City, CA). Correspondingly, the expression levels of miR-137 and mRNA were calculated relative to *U6* small nuclear RNA and *glyceraldehyde-3-phosphate dehydrogenase* (*GAPDH*). The 2^−ΔΔCt^ method was adopted to analyze the fold-changes. The sequences of the primers were as follows: *U6* (forward, 5’-CTCGCTTCGGCAGCACA-3’; reverse, 5’-AACGCTTCACGAATTTGCGT-3’), *GAPDH* (forward, 5’-GGAGCGAGATCCCTCCAAAAT-3’; reverse, 5’-GGCTGTTGTCATACTTCTCATGG-3’), miR-137 (forward, 5’-TATTGCTTAAGAATACGCGTAG-3’; reverse, 5’-AACTCCAGCAGGACCATGTGAT-3’), *LGR4* (forward, 5’-CTTTGTTTGCCATTTCCTA-3’; reverse, 5’-CTAGTGAGTTTAATAGCACTAA-3’), *RANKL* (forward, 5’-GCCAGTGGGAGATGTTAG-3’; reverse, 5’-TTAGCTGCAAGTTTTCCC-3’), *OPG* (forward, 5’-CATGAGGTTCCTGCACAGCTTC-3’; reverse, 5’-ACAGCCCAGTGACCATTCCTAGTTA-3’) and *runt-related transcription factor 2* (*RUNX2*) (forward, 5’-TGGTTACTGTCATGGCGGGTA-3’; reverse, 5’-TCTCAGATCGTTGAACCTTGCTA-3’).

*Western blotting*

Total protein was lysed with radioimmunoprecipitation assay (RIPA) lysis buffer (Sigma‐Aldrich) and 2% protease inhibitor cocktail (Roche). The protein concentration was determined by the Pierce BCA protein assay kit (Thermo Fisher Scientific). Equal amount of protein sample (50 μg) was loaded and separated by 10% sodium dodecyl sulfate-polyacrylamide gel electrophoresis and then transferred to the polyvinylidene difluoride membranes (Millipore, Bedford, MA). The membranes were blocked with 5% nonfat dry milk (BioRuler, Danbury, CT) and then incubated overnight at 4 °C with primary antibodies (1:1000) against GAPDH (Cell Signaling Technology, Beverly, MA; 5174S), LGR4 (Abcam, Cambridge, UK; ab75501), RANKL (Santa Cruz Biotechnology, Dallas, Texas; sc-377079), OPG (Santa Cruz Biotechnology; sc-11383) and RUNX2 (Cell Signaling Technology; 12556). After being incubated with the secondary antibodies (1:10,000) of horseradish peroxidase‐conjugated goat anti‐rabbit (Abcam; ab6721) at room temperature for 1 h, the protein bands were visualized by the pierce ECL plus western blotting substrate (Thermo Fisher Scientific). The optical density of the bands was analyzed with the ImageJ software (National Institutes of Health, Bethesda, Maryland) and the relative expression of protein was measured using GAPDH as an internal control.

*Dual-luciferase reporter assay*

The alignments of the target region in *LGR4* were predicted by PicTar prediction software. The 3′ untranslated region (3′ UTR) of *LGR4* mRNA, including the possible binding sites to miR-137, was amplified by PCR and cloned into pEZX‐MT06 vector (GeneCopoeia, Guangzhou, China) to generate wild-type *LGR4* (*LGR4*‐WT) luciferase reporter plasmids. The mutant-type reporter vectors of *LGR4* (*LGR4*‐MT) were formed by using a site-directed mutagenesis kit (SBS Genetech, Beijing, China). As mentioned before (Fan *et al.*, 2016, 2021; Ma *et al.*, 2020), the luciferase assay was performed by the co-transfection of *LGR4*-WT or *LGR4*-MT plasmids, NC or miR-137 mimics and lipofectamine 3000 (Invitrogen). At 48 h after transfection, the luciferase activity was examined by the dual-luciferase reporter assay system (Promega, Madison, WI) and normalized to *Renilla* luciferase activity.

*Statistical analysis*

Data were shown as mean ± standard deviation (SD) of three individual experiments and analyzed with the SPSS Statistics 20.0 software (IBM, Armonk, NY). Student’s *t*-test or one-way analysis of variance (ANOVA) followed by Tukey’s test was used to compare the differences of two or multiple groups, respectively. A P value < 0.05 was considered as statistically significant.

## Results

**MiR-137 played a negative role in the osteogenic differentiation of hASCs *in vitro***

**Figure 1 -:**
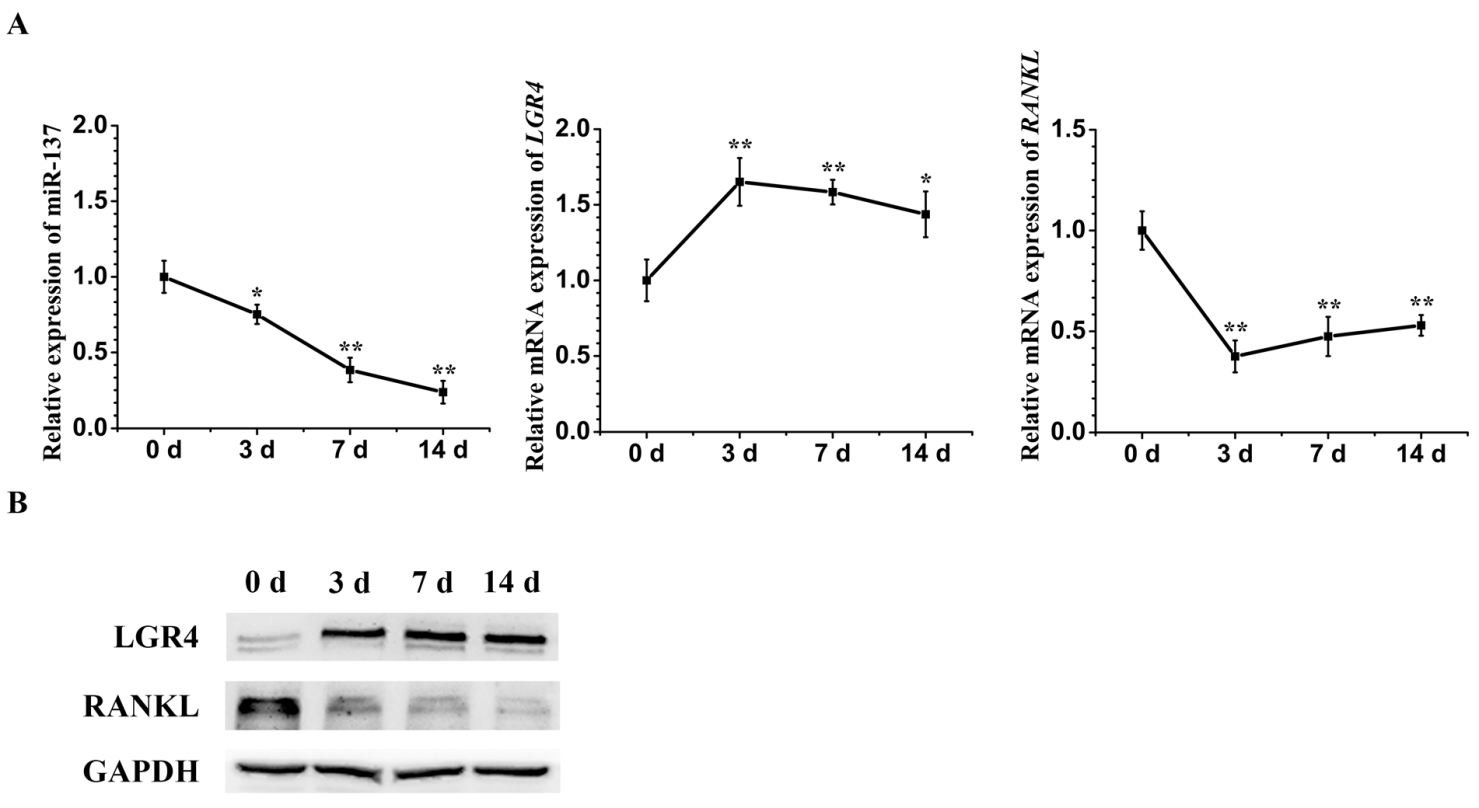
The expression characteristics of key factors in our signaling network model during the osteogenic induction of hASCs. (A) qRT-PCR analyses (0 d, 3 d, 7 d and 14 d) of miR-137, *LGR4* and *RANKL* relative mRNA expression during the osteogenic differentiation of hASCs. (B) Western blotting analyses (0 d, 3 d, 7 d and 14 d) of LGR4 and RANKL protein expression during the osteogenic differentiation of hASCs. All experiments were performed in triplicate. Data are presented as mean ± SD. *P<0.05, **P<0.01.

In order to detect the expression profile of key factors in our signaling network model during the osteogenic induction of hASCs, we detected the expression of miR-137, *LGR4* and *RANKL* at different time points (0 d, 3 d, 7 d and 14 d). We observed that the expression of miR-137 and *RANKL* decreased along with the osteogenesis of hASCs while *LGR4* increased (Figure 1). To confirm the role of miR-137 in osteogenisis, hASCs were transfected with lentivirus constructions for NC and for overexpressing or downregulating miR-137 in a transfection rate over 90% (Figure S1A). The qRT-PCR analysis showed a more than 10-fold increase of miR-137 in the miR-137 overexpression group while a decrease of about 90% in the miR-137 knockdown group (Figure S1B). After a 7-day culturing in PM or OM, the ALP activity assay of transfected hASCs displayed that miR-137 overexpression weakened the ALP activity, but the knockdown of miR-137 enhanced it (Figure S2 A and B). Similarly, after a 14-day culturing in PM or OM, the ARS test of transfected hASCs manifested that miR-137 overexpression reduced the mineralization level of extracellular matrix whereas inhibition of miR-137 significantly promoted the formation of calcified nodules (Figure S2C and D). These data authenticated that miR-137 negatively regulated the osteogenesis of hASCs *in vitro*.

**MiR-137 downregulated *LGR4* while upregulated *RANKL* during osteogenic inhibition**

**Figure 2 -:**
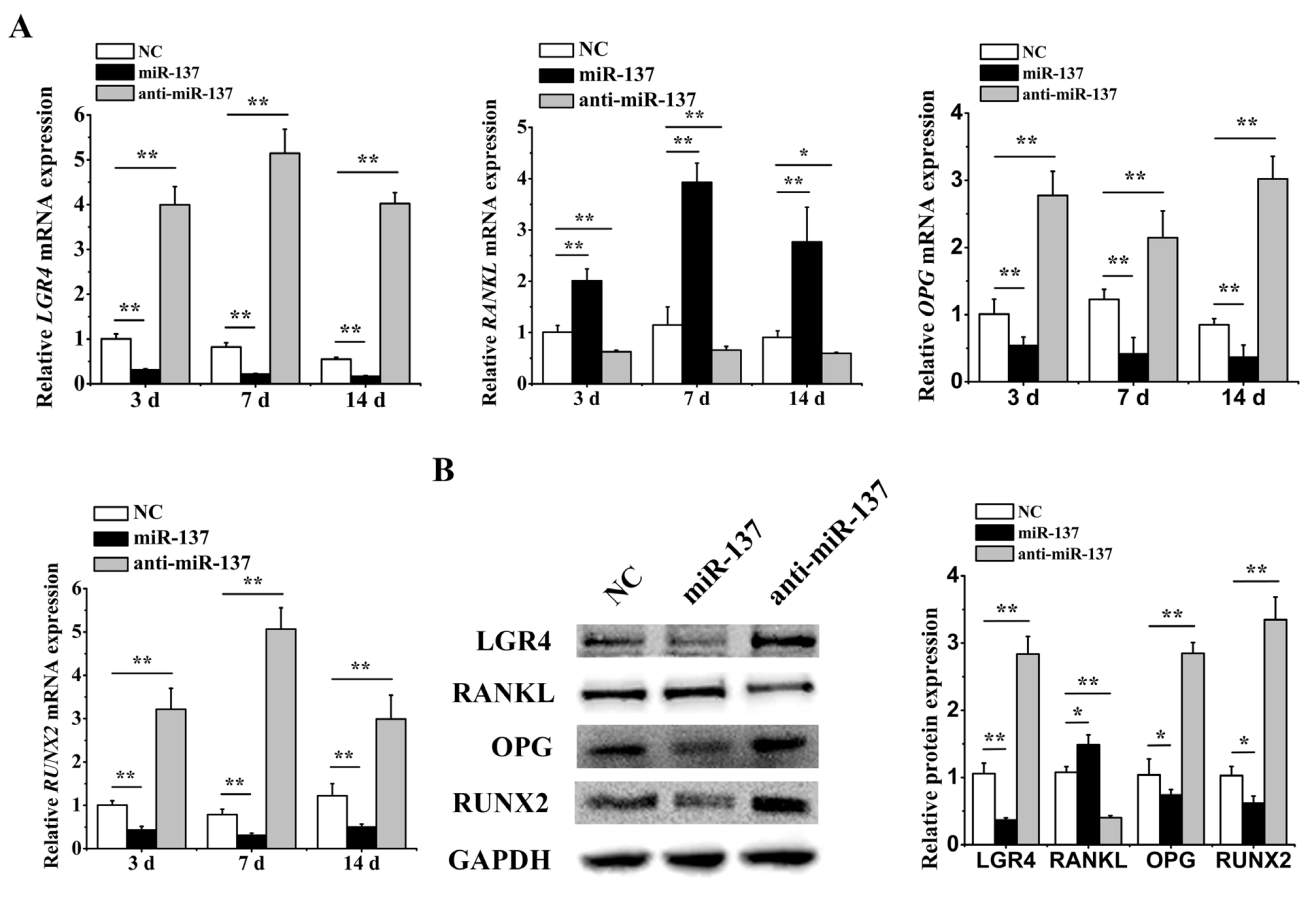
MiR-137 downregulated *LGR4* while upregulating *RANKL* during osteogenic inhibition. (A) qRT-PCR analyses (3 d, 7 d and 14 d) of *LGR4*, *RANKL*, *OPG* and *RUNX2* relative mRNA expression with miR-137 overexpression or knockdown. (B) Western blotting analyses (7 d) of LGR4, RANKL, OPG and RUNX2 protein expression with miR-137 overexpression or knockdown. All experiments were performed in triplicate. Data are presented as mean ± SD. *P<0.05, **P<0.01.

By overexpressing or knocking down miR-137 in hASCs, we first investigated the effects of miR-137 on *LGR4* expression. Both the qRT-PCR (3 d, 7 d and 14 d) and western blotting (7 d) results showed that *LGR4* was suppressed by miR-137 overexpression but activated by miR-137 knockdown. Then we further identified the impacts of miR-137 on *RANKL* and found that miR-137 positively regulated the expression of *RANKL* at mRNA and protein levels. As critical osteogenesis-related genes, both *OPG* and *RUNX2* presented opposite trends with the alterations of miR-137 (Figure 2), which was accordant with the negative effects of miR-137 on osteogenesis. These results suggested that miR-137 could suppress *LGR4* but induce *RANKL* in osteogenic impairment.

**MiR-137 directly targeted *LGR4* in hASCs**

**Figure 3 -:**
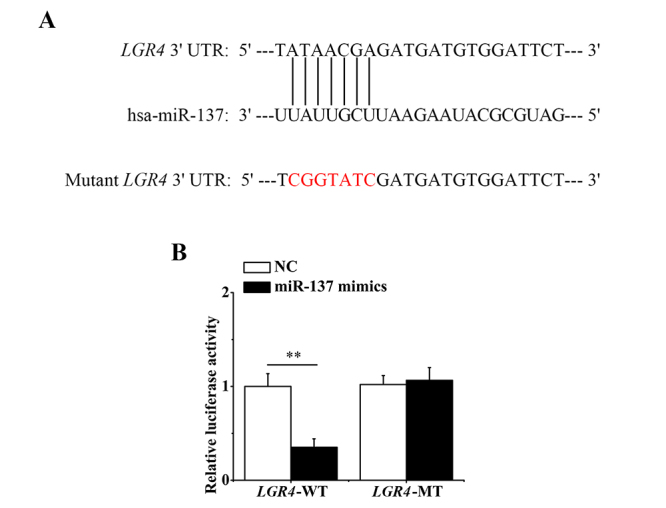
MiR-137 directly targeted *LGR4* in hASCs. (A) PicTar software predicted the binding sites of miR-137 in the 3′ UTR of *LGR4*-WT (the red sections denoted the mutated bases in *LGR4*-MT). (B) Analyses of the relative luciferase activities in the *LGR4*-WT and *LGR4*-MT group 48 h after transfection. All experiments were performed in triplicate. Data are presented as mean ± SD. **P<0.01.

To confirm whether *LGR4* is a direct target of miR-137, we performed the dual-luciferase reporter assay. The assumed targeting sites of miR-137 in the 3′ UTR of *LGR4* were predicted by PicTar software (Figure 3 A). As shown in Figure 3 B, the relative luciferase activity was significantly reduced by miR-137 mimics in the *LGR4*-WT group 48 h after transfection, whereas it was not affected in the *LGR4*-MT group. These findings support that miR-137 can target *LGR4* and downregulate its expression in hASCs.

***LGR4* knockdown attenuated osteogenesis by activating *RANKL***

**Figure 4 -:**
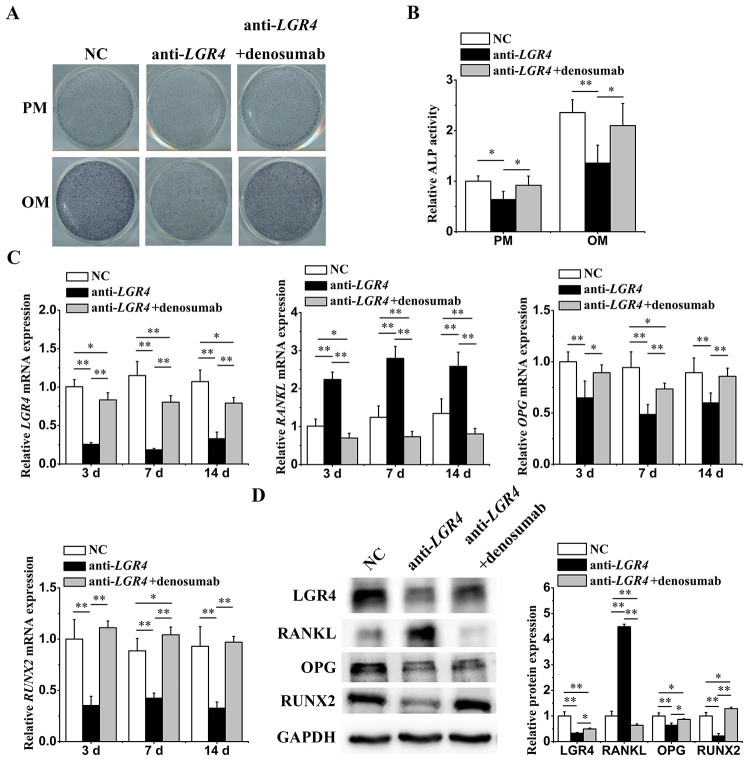
*LGR4* knockdown attenuated osteogenesis by activating *RANKL*. ALP staining (A) and quantification (B) of hASCs transfected with NC, *LGR4* knockdown lentiviruses or simultaneously treated with denosumab (*RANKL* inhibitor) for 7 days. (C) qRT-PCR analyses (3 d, 7 d and 14 d) of *LGR4*, *RANKL*, *OPG* and *RUNX2* relative mRNA expression with *LGR4* knockdown or simultaneously treated with denosumab. (D) Western blotting analyses (7 d) of LGR4, RANKL, OPG and RUNX2 protein expression with *LGR4* knockdown or simultaneously treated with denosumab. All experiments were performed in triplicate. Data are presented as mean ± SD. *P<0.05, **P<0.01.

To further clarify the involvement of *LGR4* in the osteogenic regulation of miR-137, we transfected hASCs with NC and *LGR4* knockdown lentiviruses and examined the osteogenic potential and *RANKL* expression. The ALP staining and quantification on 7 d showed that *LGR4* knockdown reduced the osteogenic ability, suggesting that *LGR4* was a positive regulator in the osteogenesis of hASCs (Figure 4 A and B). After confirming the effective silencing of *LGR4*, we observed that *LGR4* knockdown prominently elevated the mRNA (3 d, 7 d and 14 d) and protein (7 d) levels of *RANKL*. Moreover, the downregulation of *OPG* and *RUNX2* caused by *LGR4* knockdown also confirmed the inhibitory effects of *LGR4* knockdown on the osteogenic differentiation (Figure 4 C and D).

To verify whether *LGR4* could regulate osteogenesis depending on *RANKL*, we applied denosumab (*RANKL* inhibitor) to the *LGR4* knockdown group and examined the osteogenic capacity and expression of genes involved in our hypothetical signaling network (*LGR4*, *RANKL*, *OPG* and *RUNX2*). Both the ALP activity and expression of related genes showed that denosumab alleviated or even reversed the effects of *LGR4* knockdown caused in hASCs (Figure 4). Taken together, the results showed that *LGR4* knockdown impaired osteogenesis through *RANKL* stimulation and indicated the negative relationship between *LGR4* and *RANKL* during the osteogenic regulation of miR-137.

***RANKL* knockdown promoted osteogenesis by inducing *LGR4***

**Figure 5 -:**
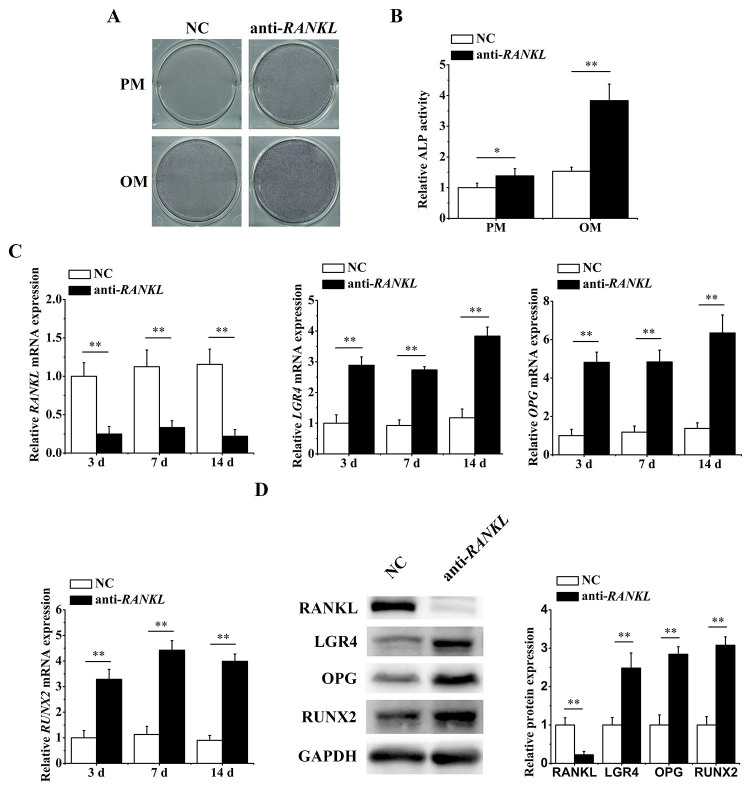
*RANKL* knockdown promoted osteogenesis by inducing *LGR4*. ALP staining (A) and quantification (B) of hASCs transfected with NC or *RANKL* knockdown lentiviruses for 7 days. (C) qRT-PCR analyses (3 d, 7 d and 14 d) of *RANKL*, *LGR4*, *OPG* and *RUNX2* relative mRNA expression with *RANKL* knockdown. (D) Western blotting analyses (7 d) of RANKL, LGR4, OPG and RUNX2 protein expression with *RANKL* knockdown. All experiments were performed in triplicate. Data are presented as mean ± SD. *P<0.05, **P<0.01.

To gain further insights into the crosstalk between *LGR4* and *RANKL*, we knocked down *RANKL* and tested whether *LGR4* and osteogenic ability were affected. Our results showed that *RANKL* knockdown led to enhanced osteogenic potential of hASCs, which was proved by the ALP activity assay on 7 d (Figure 5 A and B). After examining the expression at mRNA (3 d, 7 d and 14 d) and protein (7 d) levels, we found that knockdown of *RANKL* remarkably stimulated *LGR4*, *OPG* and *RUNX2* (Figure 5 C and D). Therefore, we verified the negative role of *RANKL* in *LGR4* and osteogenic differentiation of hASCs. Additionally, we further investigated whether *RANKL* knockdown had impacts on miR-137 and observed that the expression of miR-137 displayed a remarkable decline on 3 d, 7 d and 14 d (Figure S3). These findings, coupled with the facts that miR-137 positively regulated *RANKL*, prompted that miR-137 and *RANKL* formed a positive feedback circuit. In the light of the above results, we thoroughly corroborated that miR-137-induced negative reciprocal action between *LGR4* and *RANKL* could regulate the osteogenesis of hASCs.

## Discussion

MiR-137 has been reported to participate in the progression of various cancers (Li *et al.*, 2017; Liu *et al.*, 2017; Qin *et al.*, 2017; Luo *et al.*, 2018; Zhang *et al.*, 2018), development and maturation of neurons (Silber *et al.*, 2008; Szulwach *et al.*, 2010; Tarantino *et al.*, 2010; Sun *et al.*, 2011; Jiang *et al.*, 2013) and bone metabolism (Zheng *et al.*,2019; Kong *et al.*, 2020; Ma *et al.*, 2020; Yu *et al.*, 2020; Fan *et al.*, 2021). But compared with the previous two research fields, miR-137-modulated osteogenic differentiation is still unclear. In our previous studies, we have shown that the signaling network composed of *NOTCH1*-*HES1*, *LSD1* and *BMP2*-*SMADs* pathways contributed to the osteogenic control of hASCs by miR-137 (Ma *et al.*, 2020; Fan *et al.*,2021). However, since the osteogenic differentiation is a complex biological process which involves numerous signaling molecules, it is necessary to fully understand the underlying mechanisms of miR-137 on osteogenesis in order to develop miRNA-targeted therapy for the treatment of bone diseases. In this work, we validated that *LGR4* is a target of miR-137 in hASCs and the negative interplay of *LGR4* and *RANKL* induced by miR-137 is engaged in the osteogenic differentiation of hASCs.

Firstly, we observed the decreased expression of miR-137 and *RANKL* but increased expression of *LGR4* during the osteogenic culture of hASCs. The different expression profile of these genes suggested a potential contribution for the osteogenesis in hASCs. *LGR4* is reported to affect the osteogenic differentiation of several types of mesenchymal stem cells. By impairing the Wnt/β-catenin pathway, inhibition of *LGR4* suppresses the proliferation, migration, and odonto/osteogenic differentiation of stem cells from apical papillae (Zhou *et al.*, 2017). Deletion of *LGR4* decreases bone mass through inhibiting BMSCs differentiation to osteoblasts and delays fracture healing following by BMSCs transplantation therapy (Sun *et al.*, 2019). In hASCs, *LGR4* silencing inhibits the activity of ERK signaling and blocks the osteogenesis (Zhang *et al.*, 2017). After revalidating the inhibitory effects of miR-137 on the osteogenic differentiation of hASCs *in vitro*, we found that miR-137 was a negative regulator of *LGR4* as well. Importantly, the dual-luciferase reporter assay determined that miR-137 directly bound to the 3′ UTR of *LGR4*. So far, only two studies have reported the binding of *LGR4* by miR-137 in prostate cancer cells (Zhang *et al.*, 2020), U-2 and MC3T3 cells (Liu and Xu, 2018). Here in hASCs, we validated that *LGR4* is also a target gene of miR-137 in hASCs. To clarify whether miR-137-regulated osteogenesis relied on the direct suppression of *LGR4*, we knocked down *LGR4* and observed that both the ALP activity and osteogenic markers (*OPG* and *RUNX2*) expression were restrained while *RANKL* was induced. These findings demonstrated that miR-137 could attenuate osteogenesis of hASCs by directly targeting *LGR4*.

RANK-RANKL signaling is a canonical pathway in osteoclast proliferation and differentiation. Besides having impacts on osteoclastogenesis, RANKL is found to be involved in osteogenesis as well. RANKL promotes osteoblastic activity in human aortic smooth muscle cells by upregulating BMP2 release from human aortic endothelial cells (Davenport *et al.*, 2016). Whereas in BMSCs, RANKL regulates bone homeostasis by inhibition of osteogenic differentiation and promotion of osteoclast differentiation (Chen *et al.*, 2018b; Elango *et al.*, 2019). By overexpressing or knocking down miR-137, we found that *RANKL* was positively regulated by miR-137. To further elucidate the role of *RANKL* in the osteogenesis of hASCs, we examined relative ALP activity and expression of *OPG* and *RUNX2* to assess the osteogenic capability with *RANKL* knockdown. Our results showed that *RANKL* inhibition markedly increased ALP activity and osteogenesis-related genes expression, which were accordant with the above results that miR-137 knockdown promoted osteogenesis while downregulating *RANKL*. Consequently, *RANKL* served as a negative regulator in the osteogenic differentiation of hASCs.

Based on the above conclusions that *LGR4* and *RANKL* separately had positive and negative effects on the osteogenesis of hASCs, we further investigated the relationship between these two proteins. Recently, as a novel RANKL receptor, LGR4 is found to compete with RANK to bind RANKL and activate the Gαq/GS3K-β signaling pathway, resulting in the blockage of osteoclastogenesis (Luo *et al.*, 2016). Additionally, *LGR4* is a transcriptional target of the canonical *RANKL*-*nuclear factor of activated T cells 1* (*NFATc1*), showing that *LGR4* acts as the feedback loop controlling RANKL activities (Renema *et al.*, 2016). Nevertheless, *LGR4* silencing can prevent PTH-induced vascular calcification without changing *RANKL* and *OPG* expression in VSMCs (Carrillo-López *et al.*, 2021). So far, this is the first study to evaluate the crosstalk between *LGR4* and *RANKL* during the osteogenesis of hASCs. We have observed marked expression of *RANKL* by *LGR4* knockdown, and that the inhibition of *RANKL* relieved or even reversed the outcomes produced by *LGR4* deficiency. Therefore, these results suggest that *LGR4* knockdown attenuated the osteogenesis of hASCs by stimulating *RANKL*. Moreover, *RANKL* knockdown dramatically activated *LGR4* and osteogenic differentiation, indicating that *RANKL* had negative effects on *LGR4* and osteogenesis of hASCs. Interestingly, *RANKL* silencing led to declined expression of miR-137 and suggested that *RANKL* had positive feedback effects on miR-137, and that the downregulation of miR-137 induced by *RANKL* knockdown might further enhance *LGR4* stimulation. The involvement of miR-137-*RANKL* positive feedforward loop could partly explain the negative interrelationship between *LGR4* and *RANKL*, though the synergistic effects of *LGR4* and *RANKL* were reported in osteoclastic lineage (Luo *et al.*, 2016; Renema *et al.*, 2016). Also, given the diversity of biological characteristics of different cell lines, we speculated other signals might participate in the mediation of *LGR4*-*RANKL* crosstalk and further investigations need to be conducted.

**Figure 6 -:**
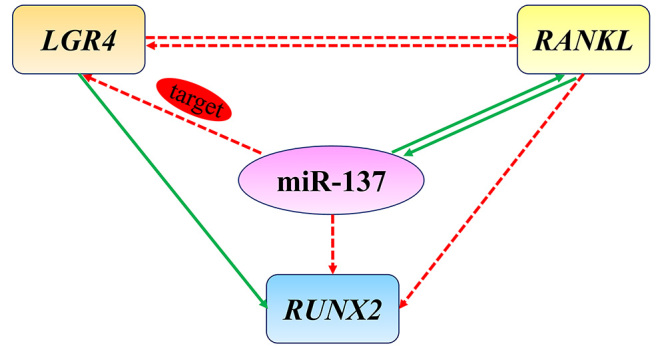
A molecular mechanism model involving the miR-137-mediated negative crosstalk between *LGR4* and *RANKL* during the osteogenic differentiation of hASCs. In brief, miR-137 directly targeted *LGR4* while positively regulated *RANKL*. Remarkably, negative interplay existed between *LGR4* and *RANKL* and could be synergistic reinforced by miR-137. The green solid lines indicated positive regulation and the red dotted lines indicated negative regulation.

Taken together, our study revealed that miR-137 negatively regulated the osteogenic differentiation of hASCs by direct binding to *LGR4* and established a molecular mechanism model elaborating the critical role of *LGR4*-*RANKL* negative interplay which was mediated by miR-137 (Figure 6). These findings provide important insights for the future of miRNA-based therapeutics in bone metabolism disorder.

## Supplementary material

The following online material is available for this article:

Figure S1 - The efficiency and effects of lentiviral transfection.

Figure S2 - MiR-137 played a negative role in the osteogenic differentiation of hASCs *in vitro*.

Figure S3 -
*RANKL* knockdown suppressed the expression of miR-137.
