# Supplementary material for: MiR-137-mediated negative relationship between LGR4 and RANKL modulated osteogenic differentiation of human adipose-derived mesenchymal stem cells
Source: Genet Mol Biol. 2022 Sep 19;45(3):e20210322. doi: 10.1590/1678-4685-GMB-2021-0332 (PMC9495020; doi:10.1590/1678-4685-GMB-2021-0332)
Supplement: Figure S1 - [file 1415-4757-GMB-45-3-e20210332-s1.pdf]

https://doi.org/10.1590/1678-4685-GMB-2021-0332

Supplementary material to "MiR-137-mediated negative relationship between *LGR4* and *RANKL* modulated osteogenic differentiation of human adipose-derived mesenchymal stem cells"

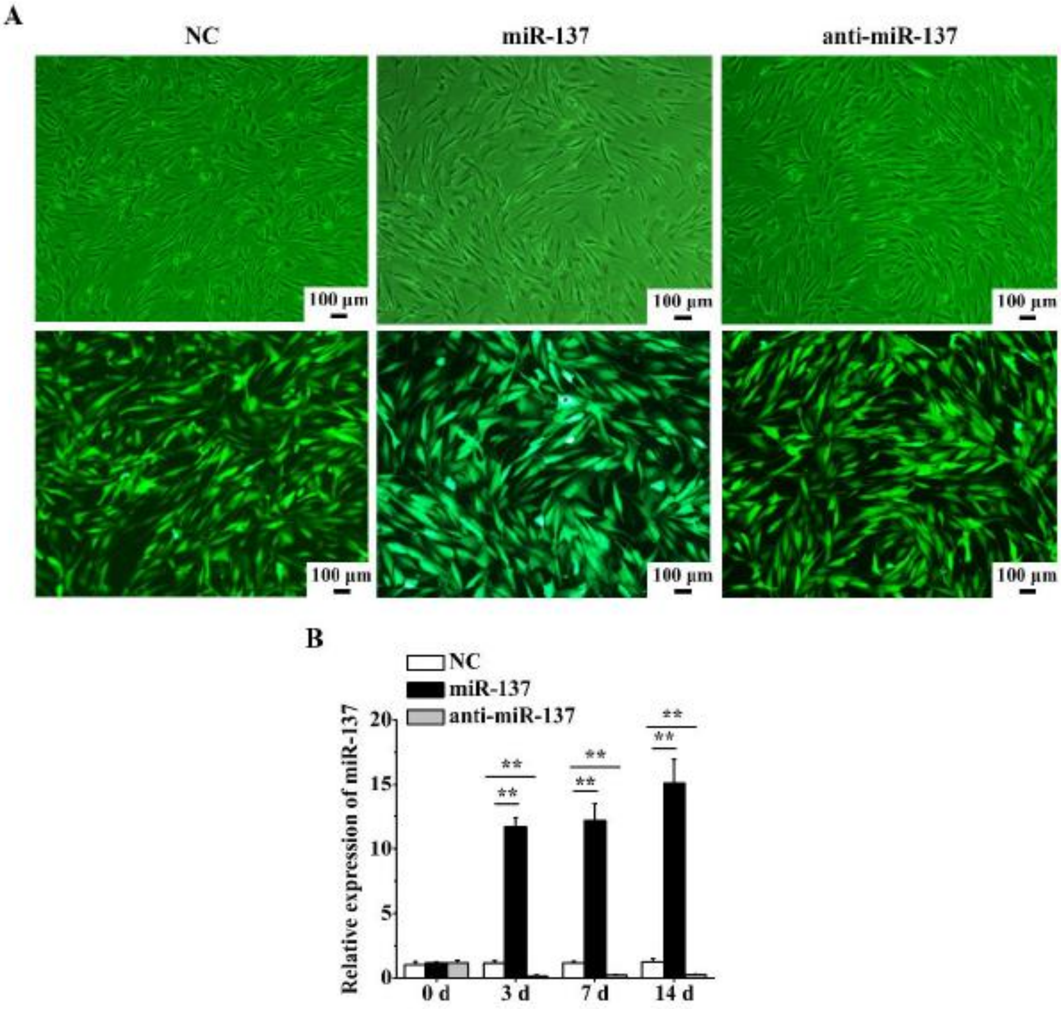

Figure S1 - The efficiency and effects of lentiviral transfection. (A) Microscopic images of GFP-positive hASCs after transfection under the ordinary (upper panel) and fluorescent light (lower panel). Scale bars: 100 µm. (B) qRT-PCR analyses of miR-137 relative expression in hASCs transfected with miR-137 overexpression or knockdown on 3, 7, and 14 days. All the experiments were performed in triplicate. Data are presented as mean ± SD. **P<0.01.
