# Supplementary material for: MiR-137-mediated negative relationship between LGR4 and RANKL modulated osteogenic differentiation of human adipose-derived mesenchymal stem cells
Source: Genet Mol Biol. 2022 Sep 19;45(3):e20210322. doi: 10.1590/1678-4685-GMB-2021-0332 (PMC9495020; doi:10.1590/1678-4685-GMB-2021-0332)
Supplement: Figure S3 - [file 1415-4757-GMB-45-3-e20210332-s3.pdf]

Genetics and Molecular Biology
https://doi.org/10.1590/1678-4685-GMB-2021-0332

Supplementary material to "MiR-137-mediated negative relationship between *LGR4* and *RANKL* modulated osteogenic differentiation of human adipose-derived mesenchymal stem cells"

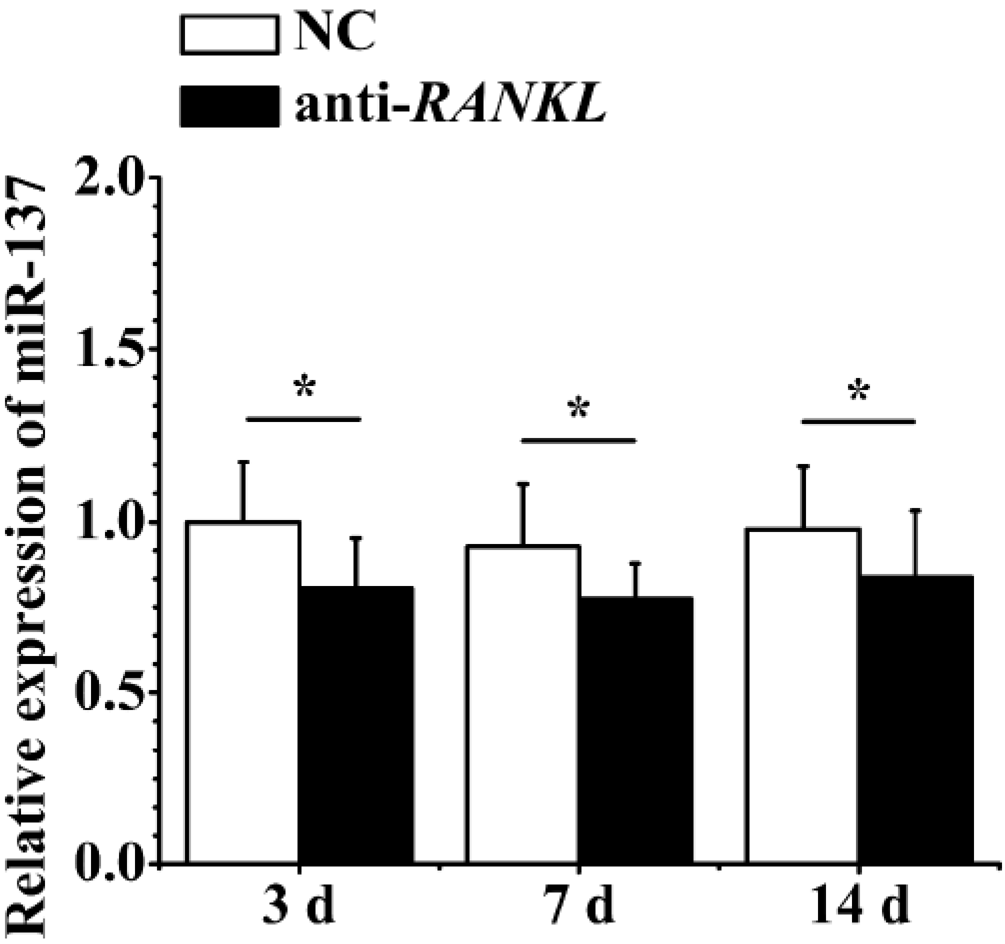

Figure S3 - *RANKL* knockdown suppressed the expression of miR-137. qRT-PCR analyses (3 d, 7 d and 14 d) of miR-137 relative mRNA expression with *RANKL* knockdown. All the experiments were performed in triplicate. Data are presented as mean ± SD. *P<0.05.
